# Validation of multiplex PCR sequencing assay of SIV

**DOI:** 10.1186/s12985-020-01473-0

**Published:** 2021-01-15

**Authors:** Ryan V. Moriarty, Nicolas Fesser, Matthew S. Sutton, Vanessa Venturi, Miles P. Davenport, Timothy Schlub, Shelby L. O’Connor

**Affiliations:** 1 Department of Pathology and Laboratory Medicine, University of Wisconsin-Madison, 555 Science Dr, Madison, WI 53711 USA; 2 Infection Analytics Program, Kirby Institute for Infection and Immunity, UNSW Sydney, Sydney, NSW 2052 Australia; 3 Faculty of Medicine and Health, Sydney School of Public Health, University of Sydney, Sydney, NSW 2000 Australia

**Keywords:** SIV, Multiplex PCR, Deep sequencing, SNV detection

## Abstract

**Background:**

The generation of accurate and reproducible viral sequence data is necessary to understand the diversity present in populations of RNA viruses isolated from clinical samples. While various sequencing methods are available, they often require high quality templates and high viral titer to ensure reliable data.

**Methods:**

We modified a multiplex PCR and sequencing approach to characterize populations of simian immunodeficiency virus (SIV) isolated from nonhuman primates. We chose this approach with the aim of reducing the number of required input templates while maintaining fidelity and sensitivity. We conducted replicate sequencing experiments using different numbers of quantified viral RNA (vRNA) or viral cDNA as input material. We performed assays with clonal SIVmac239 to detect false positives, and we mixed SIVmac239 and a variant with 24 point mutations (SIVmac239-24X) to measure variant detection sensitivity.

**Results:**

We found that utilizing a starting material of quantified viral cDNA templates had a lower rate of false positives and increased reproducibility when compared to that of quantified vRNA templates. This study identifies the importance of rigorously validating deep sequencing methods and including replicate samples when using a new method to characterize low frequency variants in a population with a small number of templates.

**Conclusions:**

Because the need to generate reproducible and accurate sequencing data from diverse viruses from low titer samples, we modified a multiplex PCR and sequencing approach to characterize SIV from populations from non-human primates. We found that increasing starting template numbers increased the reproducibility and decreased the number of false positives identified, and this was further seen when cDNA was used as a starting material. Ultimately, we highlight the importance of vigorously validating methods to prevent overinterpretation of low frequency variants in a sample.

## Introduction

Characterizing the sequence diversity of RNA virus populations is an essential component of studying viral pathogenesis and transmission in individuals [1, 2]. This sequence data can be used to identify antiviral drug resistance mutations [3], understand how viruses evolve [4, 5], and track virus transmission during epidemics [6], such as the Ebola virus outbreak in West Africa in 2014, the Zika virus outbreak in Brazil in 2015 [7, 8], and the current SARS-CoV-2 outbreak [9, 10].

The accumulation of mutations in RNA viruses can impact their pathogenesis [11]. While many mutations can be deleterious or neutral, some are beneficial for virus proliferation, survival, or transmission [4, 12]. Naturally elicited host immune responses that fail to eliminate replicating viruses select for variants that avoid immune detection [4]. Drug resistance mutations can also accumulate when antiretroviral therapy does not fully suppress virus replication [13]. Accurate detection of these variants in RNA virus populations can help determine whether therapeutic interventions eliminate or exacerbate mutations from the replicating virus population.

Sequencing RNA viruses requires the generation of viral cDNA, followed by amplification of either long (> 1000 bp) or short (< 400 bp) DNA segments. Long amplicons are used to study distantly linked nucleotides on the same virus templates using Pacific Biosciences (Brese et al. 2018) or Oxford Nanopore instruments [14, 15], In contrast, Illumina technology can generate sequence data from shorter viral segments with higher throughput, better fidelity, and improved efficiency [16]. While each approach has advantages and disadvantages, the desire to acquire sequence data with newer assays often trumps taking the time to perform experiments required to validate their sensitivity and reproducibility.

Our goal was to implement a multiplex PCR approach, similar to those used for Ebola [17], ZIKV [8], and SARS-CoV-2 [10], to improve the reproducibility and sensitivity of sequencing SIV derived from plasma with low virus titers or cell-associated vRNA isolated from different tissues. SIV dynamics are frequently studied in nonhuman primates [18–20], but the samples collected from animals with interesting biological phenotypes often have a low virus titer. With an ongoing emphasis on understanding the dynamics of SIV replication in nonhuman primates [21], we aimed to determine if the multiplex method could be applied to SIV to improve the characterization of virus populations with improved sensitivity and reproducibility.

We developed a multiplex PCR approach to amplify and sequence SIV. To validate this method, we sequenced different numbers of vRNA and viral cDNA templates of clonal SIVmac239, as well as variable ratios of two clonal SIV strains differing at 24 nucleotide positions. We found improved sensitivity and reproducibility of variant calling when normalizing to the number of viral cDNA templates added to the reaction when compared to the number of vRNA templates added to the reaction. By validating the SIV multiplex sequencing method here, we identify the strengths and limitations of this method, which are essential for defining the usability of any new technique.

## Results

### Design of a multiplex PCR assay for SIV

**Fig. 1:**
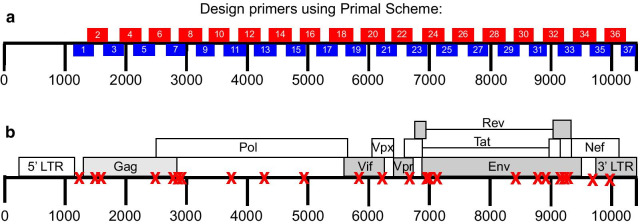
**a** SIV multiplex primer scheme. Two non-overlapping pools corresponding to even (red) and odd (blue) primer sets were designed using Primal Scheme to generate small amplicons spanning the entire SIVmac239 genome. Primer pairs were pooled at varying concentrations described in Table 1. **b** Locations of SNPs in SIVmac239-24x. SNPs denoted with a red X. SNPs are present across the entire SIVmac239 genome and are present in all genes

Candidate multiplex primers for SIV were designed in Primal Scheme, a tool developed by Quick et al. [8]. Each primer set was tested individually and then pooled such that the amplicon products would not overlap with each other Fig. 1a, Table 1. The most 5′ primer binds just upstream of the start codon for gag, and has an identical sequence to the 3′ LTR, so it is not necessary to amplify the 5′ LTR as well. Primers generated by PrimalScheme are selected based on Primer3 software, as described in Quick et al. [8]. Primer pools were tested to verify that individual primer pairs would generate amplicons spanning the entire viral genome when combined. Final primer pair concentrations, corresponding sequences, and positions relative to SIVmac239 (Accession: M33262) reference can be found in Table 1.

**Table 1:** Description of primers used in this assay, with primer set, pool, concentration, sequences of forward and reverse primers in the set, and location of primer relative to SIVmac239 (Accession: M33262)

Primer Set	Pool	Concentration (µM)	FWD sequence	REV sequence	Forward primer reference position	Reverse primer reference position
1	1	40	TCCTGAGTACGGCTGAGTGAAG	CCTTCTTTGTTCTCCAACAGGCT	1115–1137	1475–1498
2	2	30	ATTAG G CTACG ACCCAACG G AA	AATTTCCTCCTCTG CCG CTAGA	1363–1385	1711–1733
3	1	20	ACCTAGTGGTG G AAACAGGAACA	GTCCTTGTTGTGGAGCTGGTTG	1628–1651	1987–2009
4	2	20	CCATCAAG CG G CTATG CAG ATT	GCTGCATCTGTCTGTTCTGCTC	1893–1915	2246–2268
5	1	40	TGGGGTTGCAAAAATGTGTCAGA	GCTTTCTTGGTCCCCTCTGTTG	2114–2137	2476–2498
6	2	20	GCCGGGACAGAAGGCTAGATTA	TTTAG CAG ATCCACAG CTG GGT	2376–2398	2720–2742
7	1	30	AATTTCCCCATG G CTCAAG TG C	TCCTATTCCTCCTACTATTTTTGGGGT	2644–2666	2963–2990
8	2	10	TG G ATACAG GGGCTG ATG ATTCT	CCAACTGACCATCCTTTTCCATCTTT	2881–2904	3249–3275
9	1	20	GTCGCCTTAAAGCCAGGAAAGG	GAGGTATGGAGAAATATGCATCACCT	3135–3157	3465–3491
10	2	10	TAGG AATACCACACCCTG CAGG	CCCTGTCATGTTCCAGGTCTGT	3382–3404	3708–3730
11	1	40	ACATG TG CTAG AACCCTTCAG G A	CAG TCCACTG AACTTCCTCTG TT	3602–3625	3996–4019
12	2	10	AGAGAGACCTGGACAGTG AATG A	TG TG CTAATAGTCTCACTCCATTG G T	3852–3875	4213–4239
13	1	40	GAGTCAGGACAATCAGTGGTCTTAT	ATCCTGCTTTCCCTTCTTTTGACTG	4100–4125	4473–4498
14	2	10	TCTCAACACCACCGCTAGTAAGA	TACCTTTGTGTGCTGGTACCCA	4354–4377	4728–4750
15	1	15	CCTACAG AATCAG AGAGCAGGCT	ATTTG CCTG CCCATGTATAG CC	4632–4655	4949–4971
16	2	15	CCCAG AATAGTG GCCAGACAGA	CAAAG G TG TG CTCTATCCCTG C	4869–4891	5202–5224
17	1	30	ATGGTG CTAACTTTG CTTCG CA	GGTCCCTTCCACAGTTGATCTCT	5134–5156	5497–5520
18	2	30	AGGGG ATATG ACTCCAGCAG AA	CCATCCG ACCTTAAAATG GGGC	5357–5379	5742–5764
19	1	25	AG AGGTGG ATAG CAGTTCCCAC	CTTCTCCCG CTG TAAAG CAAG G	5609–5631	5959–5981
20	2	50	GGACAGATGTAACACCAAACTATGCA	TTCCCAAG ACCTTTG CCAAACC	5885–5911	6229–6251
21	1	70	GGAGAAGAGACAATAGGAGAGGCC	CATCCCATGGTTCCCTTTGTGG	6107–6131	6458–6480
22	2	10	TCCCCCTCCAG G ACTAG CATAA	TG CTTCTAG AGGGCGG TATAG C	6385–6407	6704–6726
23	1	30	TCATG CATTTCAG AGGCGG ATG	GCATTCCTCCAAGCTGGTACAC	6615–6637	6972–6994
24	2	10	TTGGG AATCAG CTG CTTATCG C	TGTCAATCCCCATCTATCTGTCTCA	6870–6892	7231–7256
25	1	25	AG TCACAG AACAGG CAATAG AGGA	TCCCTTGTTCACATACCAAATCTGC	7102–7126	7451–7476
26	2	40	AG CTGTAAATTCAACATGACAG GGTT	GTCCTATTATCCCTACCATGCCAGT	7358–7384	7749–7774
27	1	20	GGTGGTCTCTTCATGCACAAGG	TGGGATGTTTGACAATGGTCTGC	7633–7655	7955–7978
28	2	20	AAAGCAGGCATGGTGTTGGTTT	TGGAGTTACACGTGAGGTCTCC	7876–7898	8243–8265
29	1	20	CCAG AAG CCAAAG GAACAG CAT	TTGCGAGAAAACCCAAGAACCC	8113–8135	8471–8493
30	2	20	AG ATG TG AAG AG GTACACTACTG G T	CTAAACGCACATCCCCAAGCAT	8389–8414	8700–8722
31	1	15	TAG G GTCACTG CCATCG AG AAG	TCCAAGAAGCAAGGTCAAACCA	8632–8654	8921–8943
32	2	40	AGCTGGG ATG TGTTTGG CAATT	GCCAAGTCAAGAGGCGTATCAG	8879–8901	9206–9228
33	1	100	GGCAAAGAAAGAGACGGTGGAG	AAGAGAGTGAGCTCAAGCCCTT	9101–9123	9495–9517
34	2	40	CTGGAGATCTGCG ACAG AGACT	GTAATAAATCCCTTCCAGTCCCCC	9370–9392	9734–9758
35	1	40	TTGGTAG G G GTATCAGTG AGGC	CCATGCTAGAACCTCTCCCCAA	9621–9643	9971–9993
36	2	40	CAGATGAGGCACAGGAGG ATG A	ACATCAAGAAAGTGGGCGTTCC	9880–9902	10,205–10,227
37	1	40	AGGG ACTTTCCACAAG GGG ATG	TCGGTTTCCCAAAGCAGAAAGG	10,131–10,153	10,516–10,538

We first isolated vRNA from a stock of clonal SIVmac239. For Method 1, we quantified the vRNA stock and then diluted it to 10^6^ vRNA templates per reaction. Serial dilutions of quantified vRNA were converted to viral cDNA by reverse-transcription. The multiplex PCR was performed on the viral cDNA. For Method 2, we prepared total cDNA from 10^7^ vRNA templates of each stock. We then quantified the viral cDNA with a qPCR reaction specific for *gag*. The quantified viral cDNA was diluted to 10^6^ cDNA *gag* copies per reaction and the multiplex PCR was then performed. After multiplex PCR for either Method 1 or 2, 75 ng of each pool of PCR products were combined into a single tube to generate a 150 ng DNA pool containing all the generated PCR amplicons. This amplicon library was then tagged using an Illumina TruSeq kit, and sequenced on an Illumina MiSeq.

### Detection of false positives in clonal SIVmac239

We first sequenced clonal SIVmac239 to determine the frequency of false positives when using either Method 1 or 2. We used serially diluted 100% SIVmac239 vRNA or viral cDNA for this part of the project. For each replicate using Method 1, new cDNA was prepared and then multiplex PCR and sequencing were performed. These experiments were performed in triplicate. For each replicate using Method 2, the same prepared cDNA was used for all of the multiplex PCR reactions. These experiments were performed in duplicate.

FASTQ sequences were examined using a modified version of a custom pipeline previously used to analyze multiplex PCR ZIKV sequences [22], and uploaded to a Docker container in order to ensure reproducibility. Using this tool, we randomly subsampled up to 2000 reads per amplicon across each data set and mapped them to SIVmac239 (Accession: M33262), as described in the Materials and Methods. Amplification of each PCR product does not occur equally, so by subsampling up to 2000 reads, we could attempt to informatically normalize the depth of coverage, while not oversampling any one single amplicon. VarScan (https://sourceforge.net/projects/varscan/) was then used to identify nucleotides present in the virus population that were different from the reference at a frequency of 1% or greater and had a depth of coverage of at least 1800 nucleotides, or 90% of our maximum subsampled depth. SNPeff [23]was used to annotate variants and their effect on each coding sequence. Any single nucleotide variant (SNV) present at a frequency of 1% or greater and with a depth of coverage of at least 1800 nucleotides was categorized as a false positive for our analysis. These thresholds are more conservative than the 3% cutoff and 400 × coverage required by Grubaugh et al. [24].

**Fig. 2:**
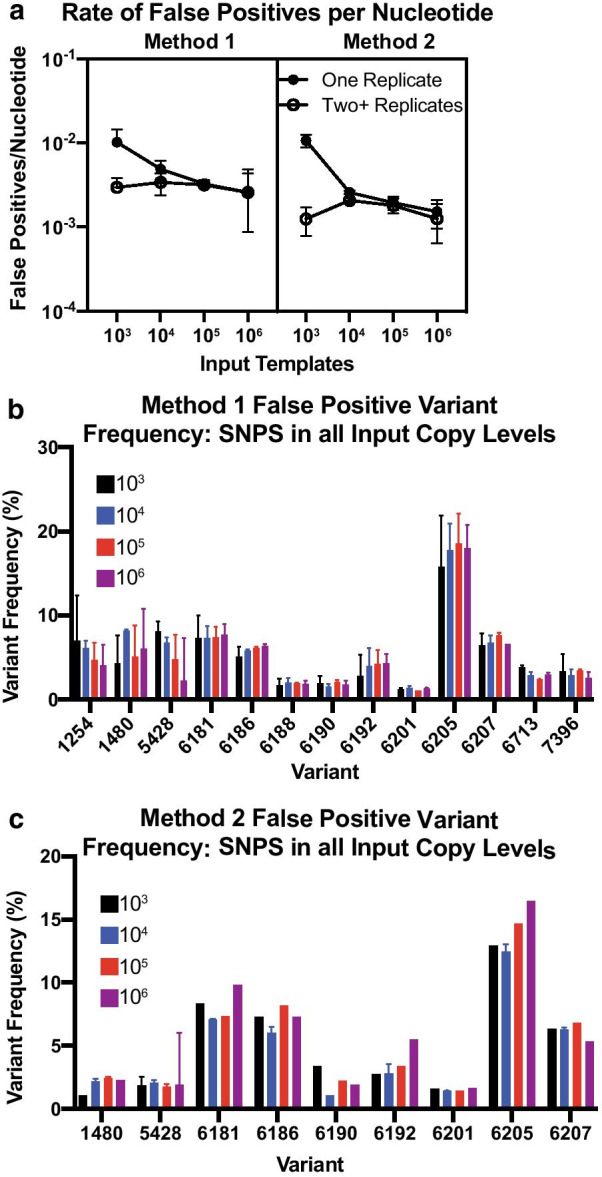
**a** Number of false positives detected per nucleotide with coverage of at least 1800 and a variant frequency of at least 1% in at least one replicate per input copy (closed circle) or at least two replicates per input copy (open circle) for our Method 1 (vRNA) (left) and Method 2 (cDNA) (right) data sets. Lines represent median ± 95% confidence interval. No significant differences were identified between data sets by Kruskal–Wallis tests. **b** False positive variant frequency of variants identified in all input templates for Method 1 data sets. **c** False positive variant frequency of variants identified in all input templates for Method 2 data sets. cDNA input template numbers denoted by colors. Lines represent mean and standard deviation for each variant’s replicate. All variants shown are present at a frequency of 1% or greater, have a nucleotide depth of at least 1800, and are detected in at least two samples

We began by assessing false positives present in sequences generated by Method 1. The average rate of false positives in a single replicate was related to the number of input templates, with samples containing 10^3^ input copies having a higher average rate of false positives at 1.13 × 10^–2^ false positives per nucleotide, and samples containing 10^6^ input copies having a lower average rate of 2.6 × 10^–3^ false positives per nucleotide Fig. 2a, left panel, closed circles, *p* < 0.0001, Tukey’s multiple comparisons test). We then determined whether the rate of false positives declined when considering two or more replicates. We found there was not a significant copy-dependent decrease in false positives when we used two replicates compared to a single replicate Fig. 2a, left panel, open circles, *p* = 0.83, Tukey’s multiple comparisons test).

We investigated the individual nucleotide positions where we detected false positives in multiple replicates when using Method 1 Fig. 2b. We found 11 positions with false positives at all input copy numbers, with 4 being insertions at nucleotide positions 1254, 1480, 5428, and 7396, and 9 being substitutions at nucleotide positions 6181, 6186, 6188, 6190, 6192, 6201, 6205, 6207, and 6713. We found the median false positive frequency did not depend on the number of input copies (*p* = 0.92, Kruskal–Wallis) Fig. 2b. Each of the insertions occurred in a poly-A region containing 6 consecutive adenines. Although the chemistry of Illumina sequencing does not lead to the same errors in homopolymers that are notorious in other sequencing platforms [25], there can still be PCR-based errors in homopolymeric regions [26, 27]. All substitutions, aside from position 6713, were present within a stretch of 27 nucleotides that are adjacent to a primer binding site. These SNVs are contained within an overlap region between Amplicons 20 and 21. Notably, these variants were present in Amplicon 21, but not Amplicon 20, suggesting that Amplicon 21 may be more prone to the incorporation of PCR-based substitutions than Amplicon 20. While unfortunate, inaccuracies in variant reporting is not an uncommon phenomenon at the ends of amplicons and has been reported previously [28, 29]. We also observed that when using a different analysis pipeline that does not normalize coverage across the genome through subsampling, these variants were not reported in the vcf file, highlighting the importance of validating the analysis methods prior to calling variants as true variants. However, we felt that the benefit of standardizing variant calling with normalized coverage across the genome outweighed the complexity associated with variabilities related to relative oversampling of individual amplicons.

We then used the same metrics to identify false positives using Method 2 Fig. 2c. Similar to Method 1, the average number of false positives per nucleotide in at least one replicate was related to the number of input templates, with 10^3^ input cDNA templates having an average of 1.05 × 10^–2^ false positives per nucleotide and 10^6^ input cDNA templates having an average of 1.52 × 10^–3^ false positives per nucleotide Fig. 2a, right panel, closed circles, *p* < 0.001, Tukey’s multiple comparisons test). When only including false positives detected in at least two replicates, there was no difference in the rate of false positives between 10^3^ and 10^6^ cDNA templates Fig. 2a, right panel).

We identified 9 nucleotide positions with false positives in at least one replicate of all input template levels using Method 2 Fig. 2c. All of the false positives detected by Method 2 were also detected by Method 1. Since these false positives are present in nearly every sample and this is a clonal virus stock Fig. 2b, c, it is likely an artifact of the method rather than true variants, highlighting the importance of validating novel methods with virus stocks of known composition. Additionally, it is important to understand the effects of nucleotide sequence and primer binding sites on false positive detection, as primer slippage may be a confounding factor.

To help determine if the rate of false positives was related to coverage depth, we calculated the frequency of nucleotide sites that had sufficient coverage (a nucleotide depth of at least 1800) for our cDNA and vRNA data sets. There was no significant difference between the percentage of bases with at least 1800 × coverage using Method 1 or 2 (Method 1 mean = 76.27% nucleotides over 1800, Method 2 mean = 75.03% nucleotides over 1800; *p* = 0.95, Mann–Whitney, data not shown), indicating that the differences in false positive frequency are more likely a result of starting template than coverage alone.

### Detection of genome-wide variants using multiplex SIV sequencing

**Fig. 3:**
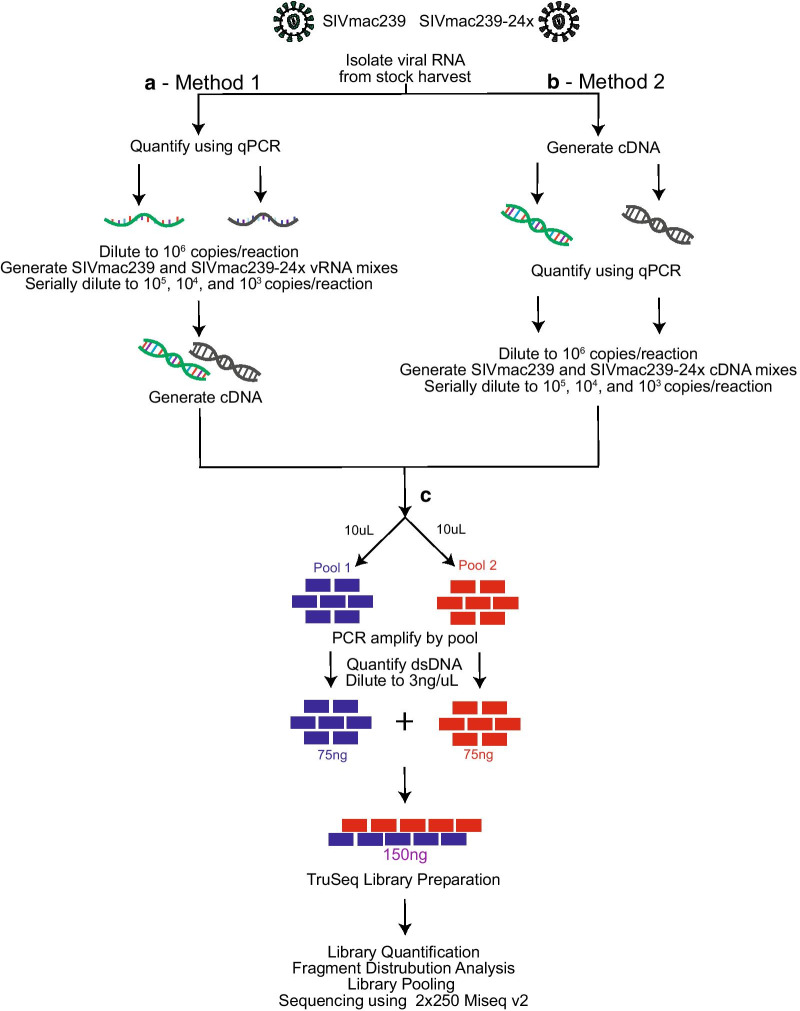
Schematic of experimental design. **a** Method 1: viral RNA was isolated from original stock and quantified via qRT-PCR. SIVmac239 and SIVmac239-24 × were diluted to 10^6^ copies/reaction and mixed at the following SIVmac239:SIVmac239-24 × ratios: 100:0, 95:5, 90:10, 75:25, 40:60, and 0:100. Serial dilutions were preformed to 10^5^, 10^4^, and 10^3^ copies per reaction. Viral cDNA was generated from viral RNA mixes, with one cDNA reaction per vRNA mix. **b** Method 2: viral RNA was isolated and approximately 10^7^ viral RNA copies were added to each cDNA synthesis reaction. Viral cDNA copies were quantified using qRT-PCR and each was diluted to 10^6^ copies per reaction. SIVmac239:SIVmac239-24 × mixes were generated at the following ratios: 100:0, 95:5, 90:10, 75:25, 50:50, and 0:100. cDNA mixes were then serially diluted to 10^5^, 10^4^, and 10^3^ copies per reaction. **c** cDNA was used for multiplex PCR. PCR products were then combined at equimolar ratios and library prepped according to TruSeq Library Preparation documentation (Illumina). Libraries were quantified, pooled, and sequenced using a 2 × 250 v2 MiSeq cartridge

We then examined the sensitivity and reproducibility of detecting individual SNVs in SIV by Methods 1 and 2. We used two stock viruses, SIVmac239 and SIVmac239-24x, that differed at 24 nucleotides throughout the entire viral coding sequence Fig. 1b, Table 2. Viral RNA was isolated from these two stocks and quantified with a *gag* qPCR assay. We proceeded with Method 1 by mixing the two stocks of vRNA to a total number of 10^6^ copies at the following SIVmac239 to SIVmac239-24 × ratios: 100:0, 95:5, 90:10, 75:25, 50:50, and 0:100 Fig. 3a. Each mixture of vRNA was serially diluted to 10^5^, 10^4^, and 10^3^ templates per 11 ul. We also tested Method 2 by first preparing viral cDNA from 10^7^ vRNA templates of each of the two stocks, quantifying viral cDNA, and then mixing the cDNA templates to a total of 10^6^ templates in the same ratios as the vRNA templates were mixed Fig. 3b. The same quantified vRNA or viral cDNA mixtures were used for the entire experiment.

**Table 2:** List of SNVs in SIVmac239-24x, with their nucleotide position, reference nucleotide, variant nucleotide, and which amplicon each SNV is present in

Position	SIVmac239	SIVmac239-24x	Amplicon
1316	T	C	1
1481	A	G	2
1510	G	A	2
2467	C	T	6
2723	A	G	7
2850	C	T	7
2860	G	A	7
3721	C	T	11
4260	G	A	13
4945	C	T	15
5815	C	A	19
6199	A	G	20
6199	A	G	21
6639	T	C	22
6639	T	C	23
6923	T	G	23
6923	T	G	24
6925	T	C	23
6925	T	C	24
7058	G	A	24
8390	G	A	29
8750	A	G	31
8850	A	G	31
9110	A	G	32
9176	A	G	32
9181	C	T	32
9642	C	T	34
9934	C	A	35
9934	C	A	36

The remaining multiplex PCR procedures were performed for the different numbers of input templates and for each of the individual ratios. PCR products were tagged, and sequencing was performed on the Illumina MiSeq. FASTQ reads were mapped to SIVmac239 and the frequencies of each individual SNV relative to SIVmac239 were determined as described for the clonal SIVmac239 data.

**Fig. 4:**
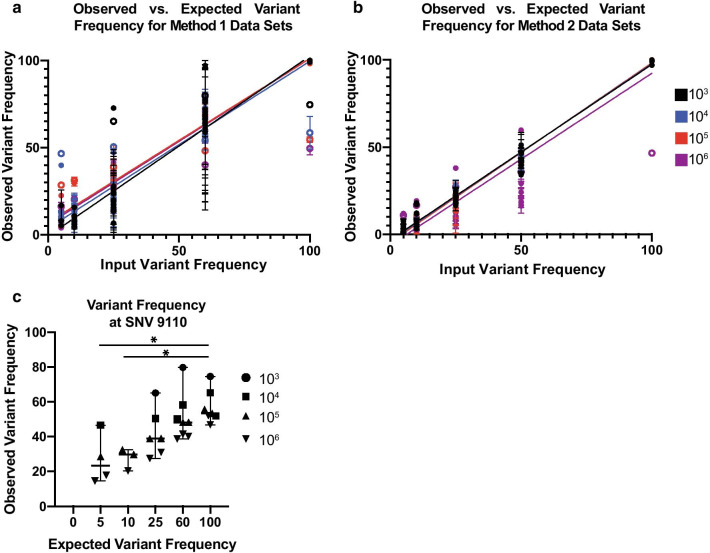
**a** Observed versus expected variant frequencies identified in the Method 1 (vRNA) mixed data sets. Observed variant frequency indicates percent SIVmac239-24× identified. Error bars indicate standard deviation for each replicate. Linear regressions colored by vRNA templates. Open circles indicate SNV 9110. **b** Observed versus expected variant frequencies identified in Method 2 (cDNA) mixed data sets. Observed variant frequency indicates percent SIVmac239-24× identified. Error bars indicate standard deviation for each replicate. Linear regressions colored by cDNA templates. Open circles indicate SNV 9110. No significant difference is observed between input templates and slope. **c** Observed versus expected variant frequency for SNV at 9110 for vRNA data sets. Lines represent medians and error bars indicate 95% confidence interval. Asterisk indicate p-value less than 0.05 as determined by Kruskal–Wallis

We compared the observed to the expected variant frequencies for all 24 positions in the genome for both Methods 1 and 2. We generated a linear regression for each number of input templates Fig. 4ato determine if the relationship between the expected and observed SNV frequency was the same. We did not find a significant difference when we compared the slopes for all four linear regression lines with either Method 1 (*p* = 0.069, Fig. 4aor Method 2 (*p* = 0.185, Fig. 4b. Notably, all of these data sets had an SNV present at position 9110 Fig. 4a, b, open circles) that was consistently detected inaccurately. While there did appear to be a slight increase in observed variant frequency when compared to expected variant frequency, site 9110 was a clear outlier in the data sets Fig. 4c.

**Fig. 5:**
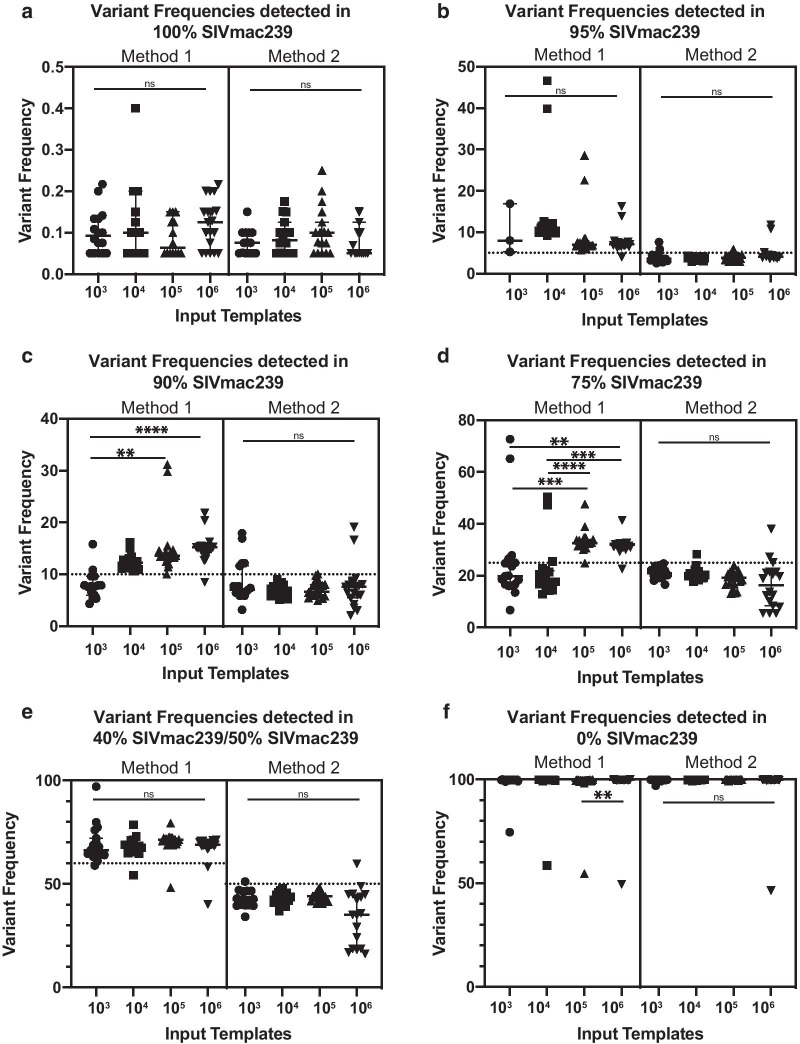
**a**–**f** Observed variant frequency of SIVmac239-24 × SNPs in varying SIVmac239:SIVmac239-24 × ratios by vRNA (left) or cDNA (right) input templates. Dotted line indicates expected variant frequency. Significance determined by Kruskal–Wallis and designated by asterisks. *p* < 0.05 (*), *p* < 0.01 (**), *p* < 0.001 (***), *p* < 0.0001 (****)

To further understand how the number of templates and the type of quantified starting material affects the reproducibility of the detected SNV frequencies, we compared the observed frequencies of each of the 24 individual SNVs across all the data sets. We found that when using Method 2, there was less variability in variant frequencies across the number of input templates when compared to using Method 1 Fig. 5a–f. This observation is consistent with data indicating that the process of reverse transcription is inefficient and variable [30], such that when 10^3^ vRNA input templates are used in the assay, it is unlikely that there are actually 10^3^ viral cDNA templates available for subsequent PCR. For both input types, it was not surprising that as the number of templates increased, the SNV frequencies tended to be more consistent and reproducible across the genome.

### Detection of variants within biological samples

To ensure the that multiplex PCR can be used with biological samples, we sequenced vRNA isolated from plasma and a lymph node (LN) of an SIV+ cynomolgus macaque at the same time point. We diluted quantified vRNA to a starting number of 10^3^, 10^4^, and 10^5^ input templates. Each sample was sequenced in triplicate. Again, we required a variant to be present at a frequency of 1% or greater and with a sample depth of 1800 to be considered a true variant. While we hoped to readily detect the same variants in sequences generated from all three numbers of input templates, we detected a substantial amount of amplicon dropout for the samples starting with 10^3^ vRNA templates. Still, we confidently obtained sufficient coverage over at least 80% of the genome from the 10^4^ and 10^5^ vRNA template data sets, and used these sets for further analysis.

**Fig. 6:**
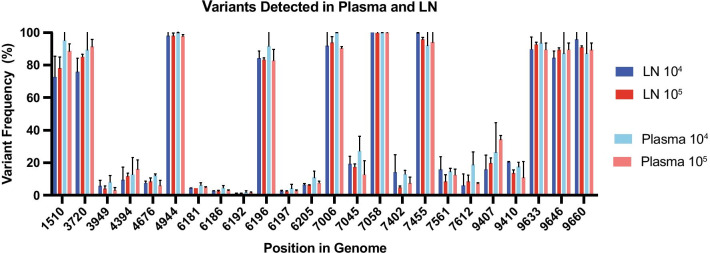
**a** Variant frequency of SNVs identified in at least two of three replicates for that input copy and sample type data set. vRNA input template numbers denoted by colors, with darker shades representing LN and lighter colors representing the plasma. Bars indicate 95% CI. All variants shown are present at a frequency of 1% or greater and have a nucleotide depth of at least 1800

We found 24 SNVs were present in at least 2 out of 3 replicates in each data set Fig. 6. However, as a result of insufficient coverage in some of the replicates, none of the variants shown in Fig. 6 were present in all three replicates of each data set. Notably, the frequencies of these 24 SNVs were similar across samples. Similar to what was shown in Fig. 2b, there was considerable variation in the nucleotide region between 6181 and 6205. Ultimately, we show that variants are detectable consistently at a minimum of 10^4^ vRNA input templates, and that variants are similar between the LN and plasma in a given animal, which is consistent with prior reports [31, 32].

## Discussion

The goal of this study was to adapt a multiplex PCR and sequencing approach [8]to sequence SIV from low quality starting material. This would include occasions where SIV is present at low titer or as partially degraded vRNA. Recognizing that different sequencing methods have their limitations, we set out to validate this approach in a series of assays described in this study.

We first sequenced clonal SIVmac239 to determine the false positive rate using both Methods 1 and 2. We found 4 nucleotide sites (1254, 2480, 5428, and 7396) in homopolymeric regions with consecutive adenines where false positive indels were detected. We predict that these insertions were introduced during the PCR step. We also found 8 individual false positives in the stock that were attributed to substitutions consistently present in the same 27-nucleotide region of Amplicon 21, but not in the adjacent Amplicon 20. We hypothesize that these substitutions are specific to the generation of Amplicon 21 and the analysis pipeline, rather than actually being real substitutions.

We also found that sequencing replicates reduced the detection of false positives, particularly when there are low numbers of input templates, consistent with previous results [24]. While we realize that there are not always enough resources available to sequence a sample in duplicate, our data highlights that caution should be taken when interpreting data from a single assay of a sample with low virus titer. Importantly, the process of validating a method with a known clonal virus stock is key to distinguishing between false positives, sequencing error, and true variants. Without doing the validation assays in this study, it would be impossible to know the benefits and technical limitations of using the multiplex PCR approach to sequence virus isolated from animals infected with SIVmac239. Detecting these method-dependent systematic errors by characterizing false positives in a clonal stock is important so that investigators using this method can perform the assay with knowledge of which variants are real and which are technical artifacts.

By mixing SIVmac239 and SIVmac239-24x, we detected variants at a frequency of 5% with as few as 1000 input copies. We opted for this conservative threshold because we already knew that there were some false positives detected when a threshold of 1% was used Fig. 2, and the most relevant variants accumulate over time to a higher frequency. Thus, detection of variants at a frequency of < 5% was less critical for broad analyses of SIV population diversity. Future studies that require more sensitive variant detection could address whether variants present between 1 and 5% can be accurately detected.

For these mixing studies, we chose two viruses with variants scattered throughout the genome, with at least one variant present in each gene. This let us determine whether we could effectively detect variants throughout the genome and across a large number of PCR amplicons generated by either Method 1 or 2. We were surprised to find it difficult to interpret the SNV frequency at position 9110. This site lies in a region dense with adenines and guanines which may contribute to some inconsistencies as a result of PCR slippage or misincorporation of nucleotides during PCR amplification [33]. In addition, the forward primer for Amplicon 33 is one nucleotide different from its complementary sequence in SIVmac239-24x due to the modified nucleotide 9110 present in the SIVmac239-24 × sequence. While we did trim primers computationally, this would not prevent PCR error from occurring. As a result, some SIVmac239-24 × templates may not be amplified as efficiently because of a single nucleotide difference, which may also lead to amplicon dropout and skewed results.

Throughout our study, we compared the results obtained using Methods 1 and 2, which used quantified vRNA and quantified viral cDNA, respectively. Reverse transcription is inefficient [30], so we wanted to determine if there were fewer false positives and more consistent detection of SNVs when quantified cDNA was used as the starting material rather than vRNA. We found the observed SNV frequencies were more similar to expected frequencies when quantified cDNA was used as a starting template Fig. 4b and, not surprisingly, when increased numbers of vRNA or cDNA templates were used. Even though our quantification of viral cDNA was based only on the copies of *gag*, we found that using quantified viral cDNA as the input improved the reproducibility of variant detection when we mixed two clonal virus inocula at predefined ratios, even when using only 10^3^ quantified templates. This observation further raises concerns that using quantified vRNA as starting material gives an overestimation of the number of vRNA templates that are actually converted to cDNA and amplified to yield the reported sequence data.

By testing this method with biological samples, we provide evidence for the use of SIV multiplex PCR in animal studies as a way to characterize viral populations in vivo. Unfortunately, we were unable to fully amplify the biological samples with the lowest input titer. This may be a consequence of host RNA or DNA that may have reduced the efficiency of primer binding. Nonetheless, we could still amplify from samples containing 10^4^ input vRNA templates isolated from plasma or host cells. We detected variants with similar frequencies that passed our threshold in two of three replicates in each data set Fig. 6. The variants that were not present in all three replicates typically did not have enough coverage in all three replicates to pass our threshold, which is stringent compared to other similar analyses [24]. Importantly, we found the variant frequencies were similar between tissues and plasma, consistent with previous reports [31, 32], and that a biologically relevant amount of virus (10^4^ total vRNA input templates) is sufficient to provide reproducible variant detection across nearly all of the SIV genome.

Overall, we found that the multiplex PCR approach could be successfully used to generate genome wide sequences of SIV, but our results strongly imply that any new sequencing and analysis methods be validated before using them widely to characterize variant frequency in a virus population. While it was possible to generate sequence data from 10^3^ vRNA templates, the use of quantified cDNA was more consistent. Further, although this method could be used to successfully detect SNVs across the genome, we found there were key features in the viral genome that affected the accuracy of the multiplex PCR approach. Thus, while the multiplex PCR method has many advantages for deep sequencing virus populations, validation experiments and visualization of the output alignments are essential for correct data reporting, as expected for any sequencing approach.

## Conclusions

Our initial goal of this study was to generate a sequencing approach that was able to characterize viral population diversity with low input templates. Multiplex PCR has been used to accurately sequence other viruses, including Zika [8], Dengue, and Chikungunya [34], at titers between 10^3^ and 10^6^ vRNA copies per mL, and most recently with SARS-CoV-2 [9, 10] and we were hoping this would extend to SIV. However, many publications fail to state the viral input titer when describing their sequencing methods. We learned that increasing numbers of input SIV templates and utilization of quantified cDNA as a starting material improved reproducibility of variant calling. Further, our data suggests that the multiplex PCR and sequencing approach may not be as sensitive at low numbers of input templates for SIV, when compared to other using low numbers of templates for other viruses. We additionally were able to demonstrate that this model may be used with biological samples and at biologically relevant levels of circulating virus. Most importantly, our study demonstrates the need to validate new sequencing approaches because the same method may not be viable for sequencing all viruses with the same sensitivity and reproducibility. We now understand the limitations of the assay so that experiments can be designed to maximize the likelihood of success and minimize the overinterpretation of data.

## Methods

### Primer design

Primers were designed using Primal Scheme, as previously described by [8]. FASTA files of SIVmac239 and three consensus sequences of virus populations isolated from animals infected with SIVmac239 were used as the foundation for the Primal Scheme tool. 37 primer pairs Table 1 were generated to span the entire SIV genome. The lengths of the resulting amplicons ranged from 285 to 397 bp, with an average length of 351 bp. The number of overlapping nucleotides for each amplicon ranged from 40 to 149 bp, with an average length of 100 bp. Primer pairs were split into two pools to ensure that the amplicons generated within each pool would not overlap. Primer sequences, pools, and concentrations can be found in Table 1. Final concentration of Pool 1 was 35 µM and Pool 2 was 24 µM.

### Isolation of vRNA for sequencing

SIVmac239 and SIVmac239-24 × vRNA were isolated from clonal virus stocks. Briefly, 1 ml of each virus stock was centrifuged at 13,000 rpm for 30 s to pellet any cells that were present. Plasma vRNA was obtained from an animal described in Ellis et al. [35] #20499, and lymph node (LN) vRNA was isolated from a necropsy tissue homogenate by adding TriZol and conducting a standard phenol–chloroform RNA extraction. The supernatant of each sample was transferred to a 1.5 mL Eppendorf tube and spun at 13,000 rpm for 1 h at 4C to concentrate virus particles. After spinning the sample, we removed all the supernatant, except 200 µL of liquid, so as not to disturb the viral pellet. The vRNA was then extracted using the Qiagen MinElute vRNA extraction kit, according to manufacturer’s protocols (Qiagen). Prior to elution, 25 µL of Buffer AVE was added directly to the MinElute Column membrane and incubated for 5 min.

### Preparation of viral cDNAs

The vRNA isolated from the SIVmac239 and SIVmac239-24 × virus stocks were each diluted to 10^6^ copies/11ul in nuclease-free water. They were mixed at SIVmac239: SIVmac239-24 × ratios of 100:0, 95:5, 90:10, 75:25, 50:50, and 0:100. These mixtures were diluted 1:10 in nuclease-free water to generate vRNA template concentration dilution series of 10^6^, 10^5^, 10^4^, and 10^3^ templates per 11uL. From each mixture, we used 11ul of vRNA and performed cDNA synthesis using SuperScript IV Reverse Transcriptase (Invitrogen), according to manufacturer’s protocol. For experiments where quantified viral cDNA was used as starting material, approximately 10^7^ viral templates were used for cDNA synthesis using SuperScript IV Reverse Transcriptase (Invitrogen), according to manufacturer’s protocol. Viral cDNA and vRNA was then quantified using a *gag* qPCR assay as previously described [36].

### Multiplex PCR reactions

Each tube of viral cDNA generated from the virus stocks or biological samples was split equally, such that 10uL of viral cDNA was PCR amplified with the two separate primer pools. Amplification was performed with the Q5 polymerase and the following reaction conditions: 98 °C for 30 s, 35 cycles of 95 °C for 15 s and 65 °C for 5 min, and then cooled to 4 °C. Products were verified using a 1% agarose gel and were quantified using the Qiagen High Sensitivity DNA kit (Thermo Fisher).

### Library preparation and sequencing

After the two amplicon pools were generated, 75 ng of each pool was mixed to generate a total of 150 ng DNA. This pool of PCR products was tagged with the Illumina TruSeq Nano HT kit, according to the manufacturer’s protocol (Illumina). Following tagging and purifying, the libraries were quantified using the Qiagen High Sensitivity DNA kit. The quality of each library was characterized with a High Sensitivity DNA kit (Agilent) on an Agilent Bioanalzyer. If unligated adapter dimers were detected at 140 bp, an additional bead clean up step was performed. The average tagged library size was approximately 503 bp (range 491–512). Tagged libraries were pooled at equimolar concentrations and diluted so that the final concentration of DNA molecules per run was 10 pM. This diluted pool and 10 pM PhiX were denatured with 0.2 N sodium hydroxide for 5 min at room temperature. Denatured PhiX was then added to the pool at a final frequency of 10 percent. Each pool was loaded at 10 pM concentration onto a 500-cycle v2 MiSeq cartridge and sequenced.

### Data analysis

FASTQ reads were demultiplexed and then processed using a modified pipeline from David O’Connor’s lab, called the Zequencer, with the initial scripts available at https://bitbucket.org/dholab/ and referenced in Dudley et al. (2017. All scripts used, and their documentation, can be found on our github repository, https://github.com/SLO-Lab/SIV_MultiplexPCR. Briefly, reads were trimmed, merged, and normalized using bbtools (https://jgi.doe.gov/data-and-tools/bbtools/) and Seqtk (https://github.com/lh3/seqtk). A FASTA file was generated that contained the nucleotide reference sequences for all 37 amplicons, as they would exist in SIVmac239 (Accession: M33262). Up to 2000 merged reads that mapped at low sensitivity to each of the 37 reference amplicons were extracted from the data set. These reads were then aligned to SIVmac239 using NovoAlign (http://www.novocraft.com/products/novoalign/). A pileup file was generated from the BAM alignment. Variants with a frequency of 1% or higher were called by VarScan (https://sourceforge.net/projects/varscan/) and annotated by SNPeff y[23]. VCF files were processed and analyzed in R(v3.6.1). Variants with a sample depth less than 1800 were discarded to reduce bias as a result of poor sample depth. Position 9609 codes for a stop codon in the *nef* protein in the M33262 Genbank reference for SIVmac239, but our stock virus is SIVmac239-nef-open, which has a T to G transversion at this position, converting the stop codon (TAA) to a glutamate (GAA) amino acid.

## Data Availability

Code used to generate data can be found on the lab’s GitHub page (see Data analysis section).

## Abbreviations

HIV: Human immunodeficiency virus
SIV: Simian immunodeficiency virus
ZIKV: Zika virus
vRNA: Viral RNA
SNV: Single nucleotide variant
